# Paraneoplastic Hypoglycemia in Hepatocarcinoma: Case Report and Literature Review

**DOI:** 10.7759/cureus.12013

**Published:** 2020-12-10

**Authors:** Carlos A Regino, Vanessa López-Montoya, Fernado López-Urbano, Jose C Alvarez, Alejandro Roman-Gonzalez

**Affiliations:** 1 Internal Medicine, University of Antioquia, Medellín, COL; 2 Internal Medicine, Faculty of Medicine, University of Antioquia, Medellín, COL; 3 Internal Medicine, Hospital Universitario San Vicente Fundación, Medellín, COL; 4 Endocrinology and Metabolism, Hospital Universitario San Vicente Fundación, Medellín, COL

**Keywords:** hypoglycemia, hepatocellular carcinoma, somatostatin, hepatitis b, therapeutic chemoembolization

## Abstract

Hypoglycemia is a common medical emergency in the context of insulin treatment in diabetic patients and oral hypoglycemic agents such as sulfonylureas. In anecdotal cases, hypoglycemia is associated with non-islet cell tumor-induced hypoglycemia (NICTH). In hepatocellular carcinoma (HCC), it has been reported in 4-27% of patients, and it is associated with poor prognosis. We present a case report of a patient with hypoglycemia associated with HCC secondary to chronic hepatitis B virus infection without response to treatment with glucagon, steroids, octreotide, and embolizations, who required parenteral nutrition at home. Even though hypoglycemia associated with HCC is a recognized entity, there is not sufficient evidence in its treatment and prevention. The article aims to review the literature on prevention and therapeutic options.

## Introduction

Hypoglycemia is a medical emergency commonly found in diabetic patients treated with either insulin or sulfonylureas. It is sporadically reported and associated with non-islet cell tumor hypoglycemia (NICTH). Plenty of mechanisms have been described: 1) Insulin-producing tumors such as pancreatic insulinomas or ectopic insulin production. 2) Tumor-mediated liver or adrenal destruction due to massive infiltration, resection, or irradiation is observed. 3) Production of molecules that interfere with glucose metabolism: cytokines, including tumor necrosis factor alpha (TNF alpha), IL-1 and 6; catecholamines (in pheochromocytomas); insulin-like growth factor I (IGF-I) and partially processed precursors of IGF-II (“big” IGF-II) secreted by tumors. 4) Hypoglycemia induced by lactic acidosis in lymphoma patients. 5) Direct consumption of glucose by tumors [1].

Hypoglycemia has been reported in up to 4-27% of hepatocellular carcinoma (HCC) patients, and it is associated with poor prognosis. HCC is associated with 23% of NICTH cases. Even though HCC-related hypoglycemia is a known phenomenon, knowledge about prevention and treatment is lacking [2].

The objective of this article is to present an HCC-associated hypoglycemia as a paraneoplastic manifestation, and therefore, to perform a literature review focused on effective treatment that is different from glucose infusion.

## Case presentation

**Figure 1:**
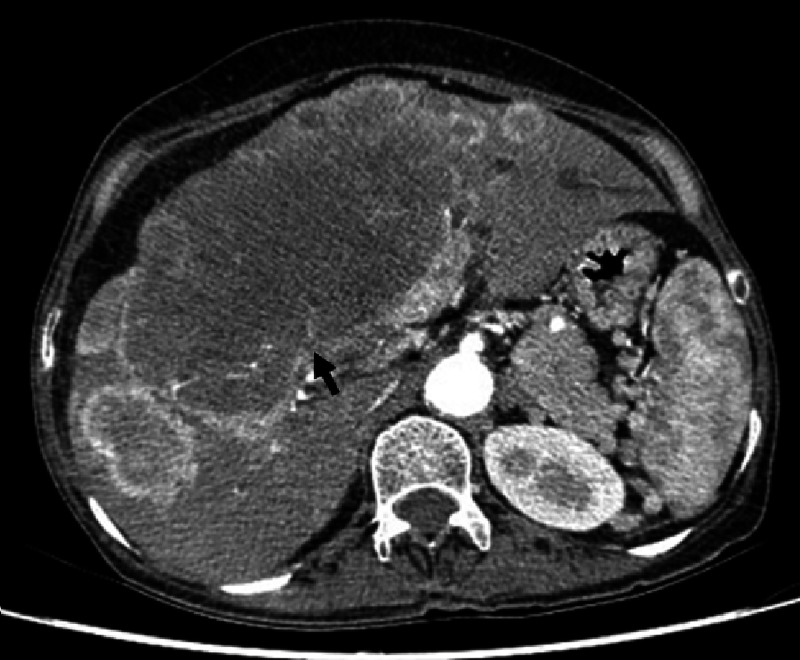
Liver MRI. Infiltrative lesions in segments IV, V, III, VII, and VIII.

A 55-year-old woman presented with a past medical history of smoking until five years ago (60 pack/year), arterial hypertension, chronic obstructive pulmonary disease with no supplementary oxygen use, and chronic hepatitis B infection with no history suggestive of cirrhosis. Her chief complaint was one year of abdominal discomfort associated with an epigastric mass and weight loss of 30 kg. Simple and contrasted abdominal computed tomography (CT) showed hepatomegaly with focal lesions, the largest being 15 cm in segment VIII, and satellite lesions of up to 5.5 cm in segment VII and 3.5 cm in segment III, with similar morphologic characteristics. Abdominal magnetic resonance imaging (MRI) showed a liver with no signs of chronic disease, and an infiltrative dominant lesion of 11 x 8 cm in segments IV and V, associated with smaller lesions in both lobules of 1.6 cm in segment III, and 0.9 cm in segments VII and VIII (Figure 1). Subsequently, the patient presented aggressiveness and disorientation, where hypoglycemia was documented (33 mg/dL) and treated with dextrose 10% with complete symptom resolution.

Physical examination revealed cachectic facies, and an abdominal mass of approximately 13 cm located in epigastrium and right hypochondrium, associated with hepatomegaly. Initial laboratory were: white blood count of 9900/uL, neutrophils 8000/uL, lymphocytes 1700/uL, monocytes 300/uL, red blood cells 5,540,000/uL, hemoglobin 16.6 g/dL, hematocrit 49.6%, mean corpuscular volume 89.6 fL, platelet count 357,000/uL, international normalised ratio (INR) 1.05 (normal range (NR) 0.8-1.4), partial thromboplastin time 33 seconds (NR 25-35), serum potassium 3.3 mmol/L, chloride 104 mmol/L (NR 98-107), sodium 141 mmol/L (NR 136-145), total bilirubin 1.01 mg/dL (NR 0.2-0.9), direct bilirubin 0.48 mg/dL (NR 0-0.3), alkaline phosphatase 404 U/L (NR 38-110), gamma-glutamyl transferase (GGT) 1017.6 U/L (NR 10-38), lactic dehydrogenase (LDH) 293 U/L (NR 120-246), alanine transaminase (ALT) 124 U/L (NR 9-52), aspartate transaminase (AST) 226 U/L (NR 14-36), serum creatinine 0.22 mg/dL (NR 0.5-0.8), serum alpha fetoprotein (AFP) 23184.1 ng/mL (NR 0.8-1), carcinoembryonic antigen 0 ng/mL, CA 19-9 antigen 26.86 U/mL (NR 0-37), cortisol 8.9 ug/dL (NR 5.27-22.45), blood ketones 1.0 mg/dL (NR < 3.5), serum glucose 17 mg/dL (NR 74-106), baseline insulin 0.40 uU/mL (NR 2.6-24.9), IGF-I < 15 ng/mL (NR 45-210), C peptide 0.0407 ng/mL (NR 1.1-4.4), IgM antibodies against hepatitis B core antigen non-reactive, antibodies against hepatitis B surface antigen < 3.10 mUI/mL (NR 0-10), antibodies against hepatitis C non-reactive, hepatitis B surface antigen (HBsAg) > 1000 (NR 0-1). Given our patient’s profile, a “big” IGF-II measurement was attempted, but the assay is not available in Colombia. Therefore, hypoglycemia due to tumor consumption vs paraneoplastic hypoglycemia due to “big”-IGF II production were the suspected diagnoses. The patient required treatment with 50% dextrose in continuous infusion to achieve normal blood glucose and to remain symptom free.

**Figure 2:**
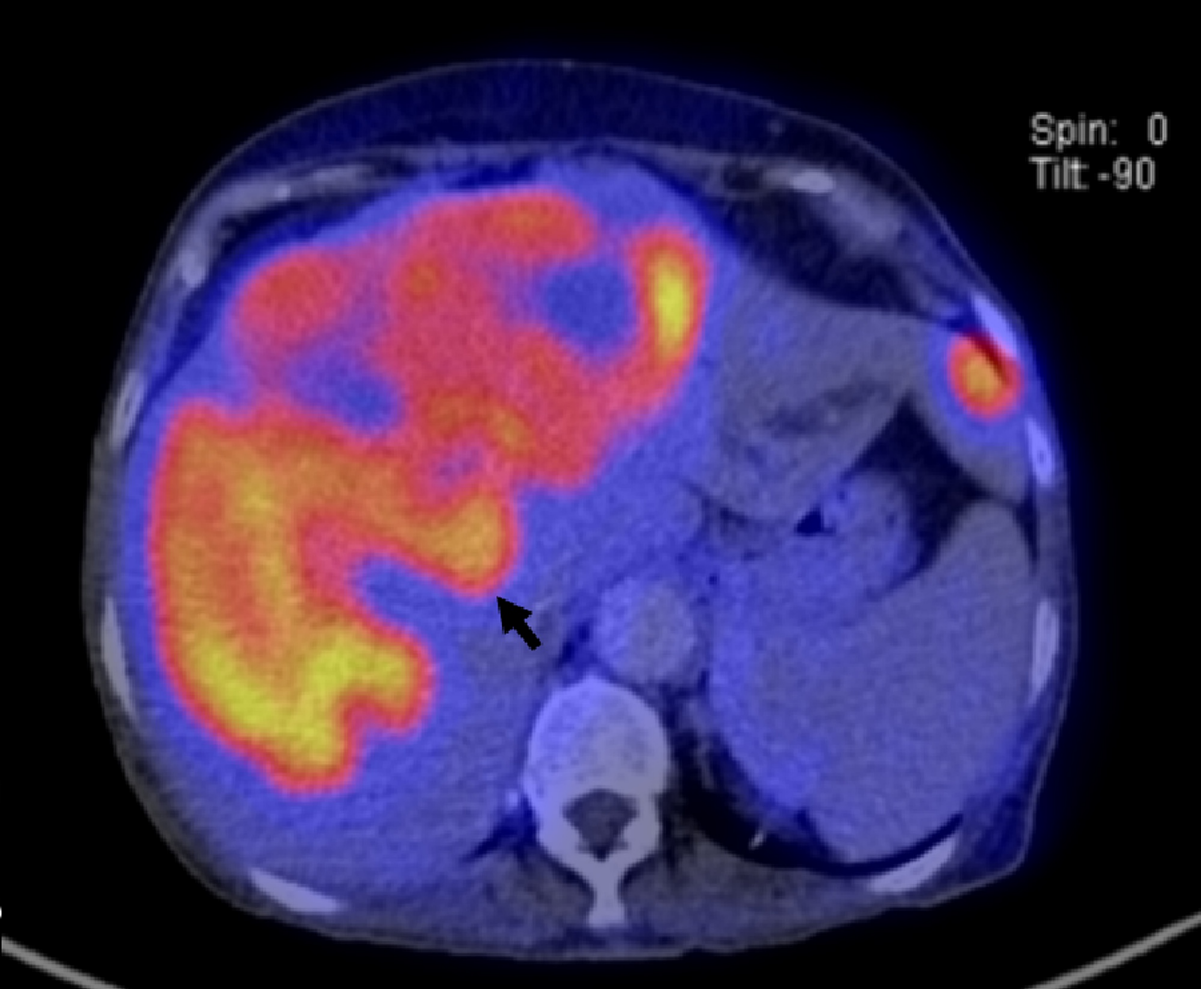
18F-FDG-PET/CT. Hypermetabolic liver lesions, with central convergence and bilateral involvement.

**Figure 3:**
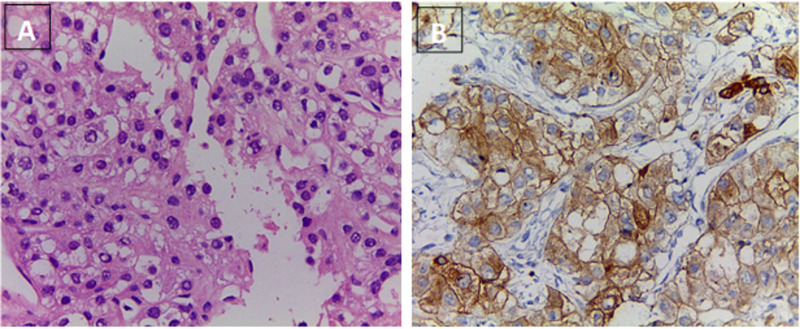
(A) Hematoxylin and eosin. Epithelial neoplasm with polygonal cells, clear cytoplasm and oval nuclei with granular chromatin and visible nucleolus. (B) Strong positive Glypican 3 immunohistochemistry in tumor cells. Images provided by the Department of Pathology, University of Antioquia.

**Figure 4:**
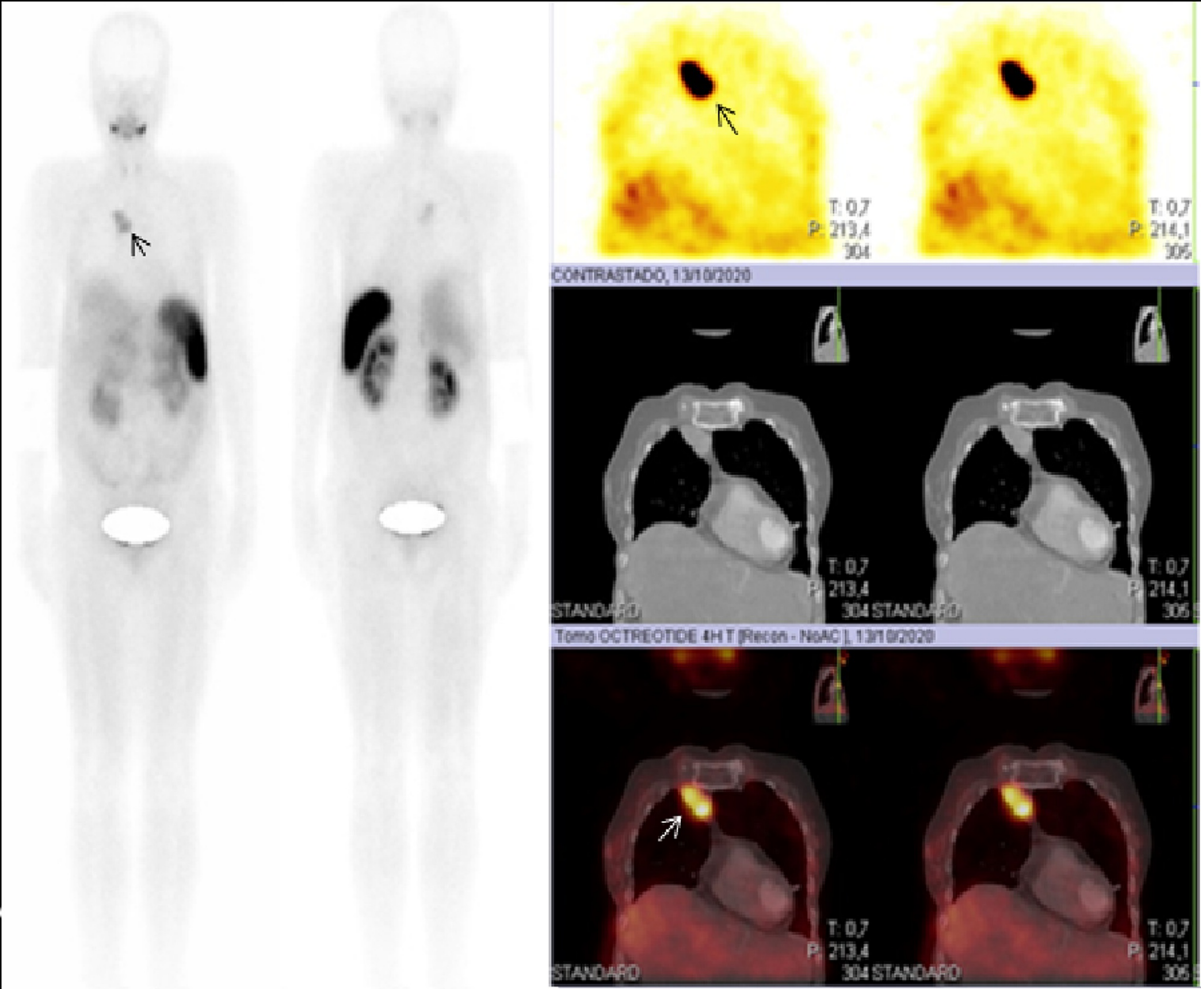
OctreoScan (also known as Somatostatin receptor scintigraphy). Positive for tumoral uptake with somatostatin receptors in thorax, with no evidence of liver uptake.

A positron emission tomography (PET/CT) with fluorine-18-fluorodeoxyglucose (18-FDG) was performed (Figure 2), where multiple hypermetabolic hepatic masses were observed, in both hepatic lobules; the high uptake index suggested an elevated glucose consumption tumor. Liver biopsy confirmed the diagnosis of trabecular and fibrolamellar hepatocarcinoma (Figure 3). Given the size and presence of metastatic disease, the patient was not a candidate for surgical treatment; transarterial chemoembolization (TACE) was done twice with partial improvement, but the dextrose infusion could not be stopped. A glucagon therapeutic test was performed, with inadequate response. Thereafter, an OctreoScan was performed (Figure 4) to determine if the patient could benefit from somatostatin analogs, but uptake was observed in a pulmonary metastasis only, reducing success probability with such therapy. Finally, despite different interventions, hypoglycemia control could not be achieved and, consequently, parenteral nutrition with a high dextrose load was started to continue ambulatory palliative care.

## Discussion

Hypoglycemia is a well-known paraneoplastic feature of HCC, occurring in up to 4-27% of patients [3]. There are two types of paraneoplastic hypoglycemia, based on the main etiologies of NICTH: type A and type B. Type A is characterized by an increased consumption of glucose by tumor in malnourished patients with low glycogen deposits and defective gluconeogenesis. This type of NICTH is observed in late-stage HCC when the tumoral burden is high and hepatic destruction is extensive [4]. Type B (less common) is caused by IGF-2 overproduction as it is partially processed by tumoral cells. This defective pro-IGF-2 crosses capillary membranes and stimulates insulin receptors more easily than normal IGF-2. It usually occurs in early stages of liver disease and is characterized by increased glucose uptake and severe hypoglycemia.

Our patient showed no signs of liver cirrhosis; low insulin and C peptide ruled out endogenous hyperinsulinism, hence, an NICTH was suspected. Even though IGF-II and “big” IGF-II levels were not available, 18F-FDG-PET/CT showed a significant rise in tumor metabolism, suggesting that the high tumor burden/metabolism caused hypoglycemia, instead of abnormal IGF-II secretion - in this case, glucose uptake would predominate in skeletal muscle, a finding previously reported [5].

The treatment priority in NICTH is tumor resection, as this leads to hypoglycemia resolution [6]. However, despite the large benefits from such therapy, it may be difficult or even impossible to perform, as big tumors are usually unresectable and patients are too ill to undergo a surgical procedure.

Patients without surgical criteria may undergo different treatment options: percutaneous ethanol injection and TACE [3]. TACE has shown favorable results, with a six-month survival improvement [7]. Despite such evidence, our patient had partial improvement, probably due to a high tumor burden. TACE has been used to treat paraneoplastic hypoglycemia primarily in metastatic insulinoma [3].

Most patients are treated with intravenous glucose infusions; despite this, response rates are bad, and second-line therapy is usually required [4].

Steroids were the most used drug to treat HCC-associated hypoglycemia. The therapeutic effect is based on hepatic gluconeogenesis stimulation and peripheral glucose intake inhibition. Furthermore, steroids can reduce “big” IGF-2 levels, whether it is through a reduction of production, or promoting a maturation process of pro-IGF-2 and normal complex formation [8]. Nonetheless, results have been mixed. Dexamethasone at a 2 mg/day associated with a midnight meal was superior to short action steroids; however, this effect was transitory [8].

Systemic chemotherapy, such as FOLFOX (oxaliplatin, 5-Fluorouracil, and leucovorin), has also been shown to be effective [3].

Besides, different drugs that directly inhibit IGF signals (PI3K-AKT-TOR or RAF-MEK-ERK) are being tested [9]. Case reports have documented the use of glucagon, growth hormone, and octreotide, however, effects are limited and provisional [10].

Octreotide has been successfully used in multiple cases (although no report of HCC-associated hypoglycemia): one of the cases was a patient with a solitary fibrous pleural tumor that required high octreotide doses plus steroids with a mild glycemic response [11].

Table 1 summarizes the different therapeutic interventions and its outcomes in patients with paraneoplastic hypoglycemia secondary to HCC.

**Table 1:** Published case reports of hepatocellular carcinoma (HCC)-associated hypoglycemia. * All patients received glucose and special diet ** M: male, F: female ¥ Sex not revealed

Author, year, reference	Age (years) and sex**	Hypoglycemia treatment*	Treatment response
McFadzean and Yeung, 1956 [12]	58, 60, 62¥	Cortisone 200 mg/d	Cortisone had no effect. In two patients, glycemic levels increased, but withdrawal led to hypoglycemia.
Schonfeld et al., 1961 [13]	27/M	Methylprednisolone 60 mg/d	No effect
Wing et al., 1991 [10]	30/M	Intramuscular growth hormone 8U q8H for two days	After 30 min glucose infusion was closed, serum glucose change was -50 mg/dL; 60 min after growth hormone therapy or prednisolone, serum glucose change was attenuated to -22 mg/dL.
Yonei et al., 1992 [14]	62/M	Glucagon, prednisolone or chemotherapy (adriamycin and cisplatin)	No effect or transitory effect
Saigal et al., 1998 [3]	24/F	Weekly ethanol injection for three weeks	Hypoglycemia crisis became infrequent, and intravenous glucose requirements were significantly reduced
Thipaporn et al., 2005 [8]	36/M	Prednisolone 40 mg/d PO followed by dexamethasone 2 mg/d	In a month follow-up, no hypoglycemia when dexamethasone was associated with a mid-night meal
Nikeghbalian et al., 2006 [15]	77/M	Complete surgical excision	Resolution of hypoglycemia
Kampitak, 2008 [16]	16/M	Systemic chemotherapy with doxorubicin	Temporary hypoglycemia control
Matsuyama et al., 2011 [17]	69/M	Dexamethasone	No positive effect
Whitsett et al., 2013 [7]	68/F	Prednisone, glucagon and transarterial chemoembolization	Dextrose infusion was reduced 50% in two weeks. Patient refuses further treatment and dies a month later due to cancer progression.
Huang and Chang, 2016 [18]	54/M	Prednisolone, glucagon and FOLFOX chemotherapy	After three months chemotherapy, hypoglycemia episodes declined
Van den Berg and Krol, 2017 [19]	87/M	Prednisolone	Hypoglycemia improvement
Yu et al., 2020 [20]	62/M	Prednisolone 60 mg/d PO	Severe hypoglycemia episodes were reduced. Pneumonia subsequently developed and control was not possible.

## Conclusions

In patients with HCC-associated hypoglycemia, once cirrhosis and liver failure are ruled out, NICTH must be considered. Even though it is a rare condition, it has high mortality and morbidity and determines a poor prognosis. Multiple treatment options are available, and surgical ones are the most effective when they are possible to be performed. Medical treatment is based on high dose IV dextrose associated with steroids, somatostatin analogs, and growth hormone, with high rates of treatment failure. Knowledge of this entity may lead to early recognition in at-risk population, and early-stage interventions to prevent further complications.
